# An on-chip imaging droplet-sorting system: a real-time shape recognition method to screen target cells in droplets with single cell resolution

**DOI:** 10.1038/srep40072

**Published:** 2017-01-06

**Authors:** Mathias Girault, Hyonchol Kim, Hisayuki Arakawa, Kenji Matsuura, Masao Odaka, Akihiro Hattori, Hideyuki Terazono, Kenji Yasuda

**Affiliations:** 1 French National Center for Scientific Research, Bordeaux, CRPP, UPR 8641, Pessac, 33600, France; 2 Biomedical Research Institute, National Institute of Advanced Industrial Science and Technology, Tsukuba 305-8565, Ibaraki, Japan; 3 Department of Ocean Sciences, Tokyo University of Marine Science and Technology, Tokyo 108-8477, Japan; 4 Waseda Bioscience Research Institute in Singapore (WABIOS), 11 Biopolis Way, Helios 138667, Singapore/3-4-1 Okubo, Shinjuku-ku, Tokyo 169-8555, Japan; 5 Department of Physics, Graduate School of Advanced Science and Engineering, Waseda University, Tokyo 169-8555, Japan

## Abstract

A microfluidic on-chip imaging cell sorter has several advantages over conventional cell sorting methods, especially to identify cells with complex morphologies such as clusters. One of the remaining problems is how to efficiently discriminate targets at the species level without labelling. Hence, we developed a label-free microfluidic droplet-sorting system based on image recognition of cells in droplets. To test the applicability of this method, a mixture of two plankton species with different morphologies (*Dunaliella tertiolecta* and *Phaeodactylum tricornutum*) were successfully identified and discriminated at a rate of 10 Hz. We also examined the ability to detect the number of objects encapsulated in a droplet. Single cell droplets sorted into collection channels showed 91 ± 4.5% and 90 ± 3.8% accuracy for *D. tertiolecta* and *P. tricornutum*, respectively. Because we used image recognition to confirm single cell droplets, we achieved highly accurate single cell sorting. The results indicate that the integrated method of droplet imaging cell sorting can provide a complementary sorting approach capable of isolating single target cells from a mixture of cells with high accuracy without any staining.

Recent advances in microfluidic devices have provided important advantages to work with small volumes compartmentalised in droplets. Droplets can include and isolate reagents, particles, cells, or multicellular organisms^1^. Moreover, a droplet has advantages to control and maintain cells within it, which allows effective handling for further processes such as separation and reactions. Encapsulation of a desired number of cells in a droplet is mainly described as a random process following Poisson’s distribution^2^. Although efficient ordination techniques for cell encapsulation have been reported in the literature, subsequent reactions are often needed to isolate droplets of interest^3^,^4^,^5^,^6^,^7^,^8^. In most studies, selection of a single droplet of interest is achieved by an active separation method^9^,^10^,^11^,^12^,^13^,^14^,^15^,^16^,^17^,^18^ [Supplementary material (SM) 1]. These droplet-sorting techniques are based on fluorescence-activated target recognition, i.e., a difference in fluorescence intensity between stained and unstained droplets. Therefore, these methods only work with labelled particles or pigmented cells, and are suitable for biological assays on the condition that they have proper fluorescent biomarkers. Hence, it is difficult to discriminate two pigmented cells with the same emission wavelength or to distinguish the difference between two colour-labelled single cells and a mixture of different single colour-labelled cells. This limitation is particularly important for marine samples in which the pigment content is well known to be a poor discriminating factor for microplankton species.

Detection of an unlabelled object encapsulated in a droplet requires an appropriate image-processing algorithm to successfully recognise target cells inside the droplet with single cell resolution. Current imaging microfluidic systems locate a target cell in an image using either a threshold value for intensity or compare the image captured from the video with a standard image^19^,^20^,^21^. Despite the obvious success of imaging microfluidic systems for a wide range of applications, droplet interrogation is mostly restricted to simple morphological information such as area, perimeter, and major/minor axis lengths.

Here, we have developed an original imaging cell sorting method capable of sorting droplets of interest depending on the morphology and number of cells encapsulated within them. This flexible method is based on a combination of software with both cell recognition and sorting algorithms and hardware. The hardware consists of a droplet image recognition setup, microfluidic controller, and consumable microfluidic chip with liquid electrodes for effective droplet sorting to reduce both fabrication time and cost. The latter application integrated the following steps in a single microfluidic chip: (*i*) encapsulation of live planktons in a droplet; (*ii*) searching for cells in the droplet and identifying its boundary in each frame captured by a high speed camera; (*iii*) identifying the cells based on the morphological characteristics of targets; (*iv*) sorting the droplet of interest for collection using direct current pulses applied to the unique electrode design, (*v*) storing the target droplets in a collection reservoir in the chip for incubation experiments.

To demonstrate the applicability of the hardware setup and software algorithm, we examined a mixture of two microplanktons with different morphologies: *Dunaliella tertiolecta* (chlorophyceae) and *Phaeodactylum tricornutum* (diatom). Then, the collected cells were incubated to examine potential damage caused by the sorting procedures by checking the difference in growth rates between the droplet-based system and a large flask incubation system. These 24-h incubations provide experimental conditions to test the effect of nanoliter incubation chambers on the growth rate of planktons.

## Results

### Controlling the droplet formation and path

**Figure 1:**
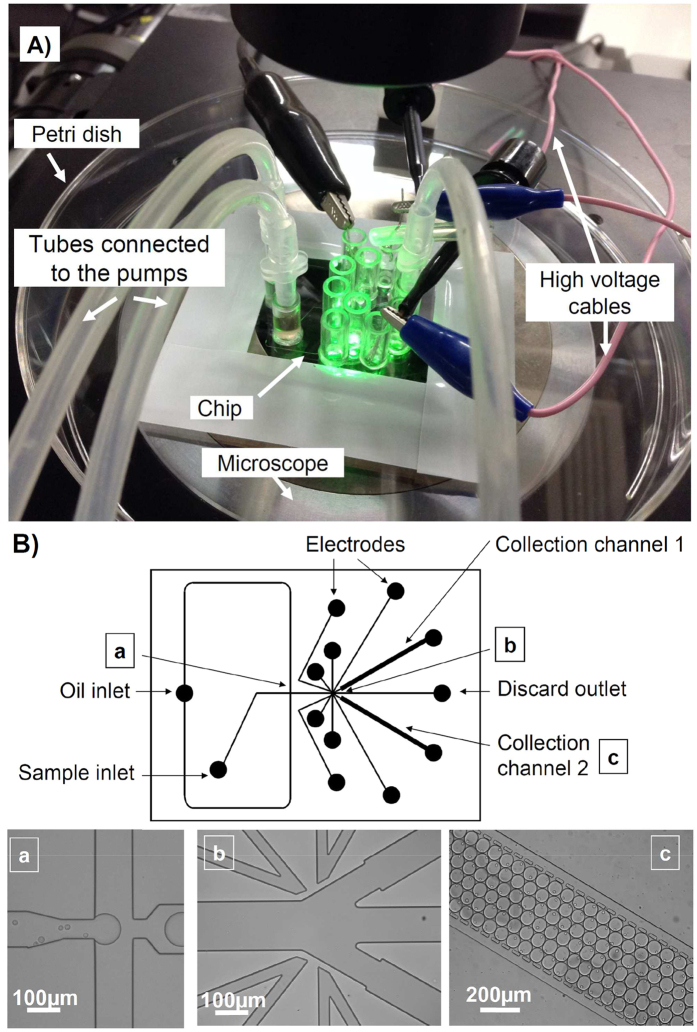
Details of the chip used for dual sorting and incubation experiments. The droplet is generated by flow focussing a sample stream with two streams of oil containing a surfactant (**a**). Image (**b**) shows both detection and sorting areas. The detection area used for recognition of targets is located in the widest channel. The sorting area consists of a discard channel for unwanted droplets and two collection channels to discriminate targets depending on the image processing result. The four narrow and sharp channels show the path of the liquid electrode integrated into the chip. Image (**c**) shows a part of the droplet trap (collection channel 2). Each collection channel can contain about 300 droplets of 1 nL suitable for biological assays.

A series of seamless direct microfluidic procedures from cell encapsulation with droplet formation, cell detection, to droplet sorting was fabricated in a chip. Because all procedures were directly connected, it reduced potential loss of the samples, production costs, time of analysis, and contamination. However, this approach needed a suitable chip design in which each channel worked together in a range of stable and strictly controlled flow velocities. In this study, monodispersed droplets were generated by flow focussing the sample stream with two streams of fluorinated oil containing a surfactant (Fig. 1). In contrast to conventional flow-focussing designs, the width of the sample channels was narrower before the flow-focussing area^10^,^22^,^23^. This choice was made to limit the back flow of oil in the sample channel when pressure applied to the oil channel was 2-fold higher than the pressure on the sample. The droplet size and frequency depending on the sample and oil pressures are shown in SM2. Six flow patterns named sample only, annular flow, plug, unstable droplet, stable droplet, and oil only were also identified depending on the sample and oil pressures^24^,^25^ (SM2). By combining SM2(a) and (b), a desired droplet size and frequency were obtained by adjusting the pressures applied to the sample and oil channels. Typically, an oil pressure of 1.5-fold higher than the sample pressure was suitable to create round droplets, enabling deflection in the collection channel. During the sorting experiment, the sample and oil pressures were limited to 10 and 15 KPa, respectively, to maintain the droplets captured in the traps [Fig. 1(c)]. The combination of droplet size and frequency also allowed optimisation of a suitable flow volume to encapsulate flagellate cells depending on their swimming speeds. The swimming speed of *D. tertiolecta* was then measured in the sample channel. The results indicated that the majority of swimming speeds were lower than 120 μm s^−1^ (2,500 measurements; SM3). These results suggested that the generation of droplet flow at a frequency of 3 Hz and diameter of 75 μm was sufficient to encapsulate most *D. tertiolecta* cells. These values could be reached in the range of working pressures of our microfluidic system (SM2). According to the integrated microfluidic design, additional oil channels used to increase the space between each droplet were not included in our chip design^9^,^26^. These empty spaces were occupied by a total of four small channels filled with saline [0.5 M NaCl) [Fig. 1(b)]. These four channels connected at the high voltage amplifier deflected droplets of interest into the collection channels by electrophoresis^27^,^28^. Our method was adapted from agarose gel liquid electrodes^19^. To reduce the viscosity for filling narrow liquid electrode spaces, we adopted saline instead of agarose gel and used simple disposable syringes (10 mL) to fill them in the narrow channel (20 μm) of thin poly(dimethylsiloxane) (PDMS). Consequently, the width of the saline liquid electrode channels could be narrower and the design sharper than agarose electrodes, leading to a concentration of electric field in a smaller area.

### Sorting cells depending on their morphologies

**Figure 2:**
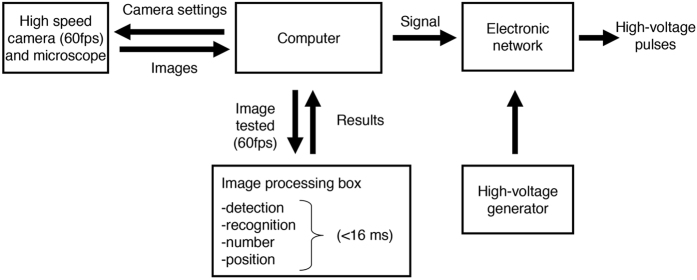
Schematic representation of the communication path between hardware. The high speed camera mounted on a microscope is connected to a computer. The computer controls both the data flow and camera settings in real-time to optimise the image processing. Each image stored in the random access memory is processed using a template-matching algorithm^30^,^31^. Depending on the desired conditions (e.g. presence of a target), a transistor-transistor logic signal is sent to an electronic network. The electronic network acts as a high speed switch device and delivers tunable short length pulses of high direct current voltage to a specific platinum electrode. The specific platinum electrode is connected to a chip channel filled with saline (0.5 M NaCl). Finally, the high voltage pulse sorts a droplet containing the target identified by the image-processing algorithm.

The present microfluidic sorting system was mainly built around the speed of image processing algorithms dedicated to search for a targeted morphology in each image captured by the high speed camera (Fig. 2). To detect cell morphology in the flow of images, we used a combination of two algorithms^29^,^30^. The first algorithm summarised the cell information (i.e. position of edges at a pixel level) and output a template image. A multiple template-matching algorithm was then developed to decrease the processing time of each image to less than 16 ms (60 frames per second)^30^. In this study, two additional steps were added to enhance the recognition and limit false detection of multiple targets in an image. The first method solved multiple detections of the same single target by merging the location of the center of templates separated by a short distance (<10 pixels). The second optimisation was keeping the maximum number of targets identified in the flow of images in the memory during a fixed time. These improvements prevented the detection of a positive single target in a droplet when a droplet was not entirely located in the detection area. In some aspects, this method is close to the single cell mode detection of conventional flow cytometry^31^.

**Figure 3:**
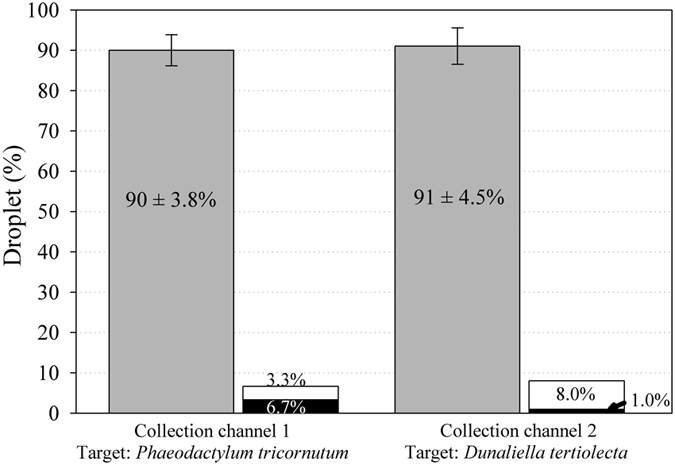
Measurement of the sorting efficiency of two plankton species: *Phaeodactylum tricornutum* and *Dunaliella tertiolecta* (n = 6). The gray bars show the percentage of droplets containing a single target in the correct collection channel. The black and white bars show false-positive errors (false target and false number of cells, respectively).

**Figure 4:**
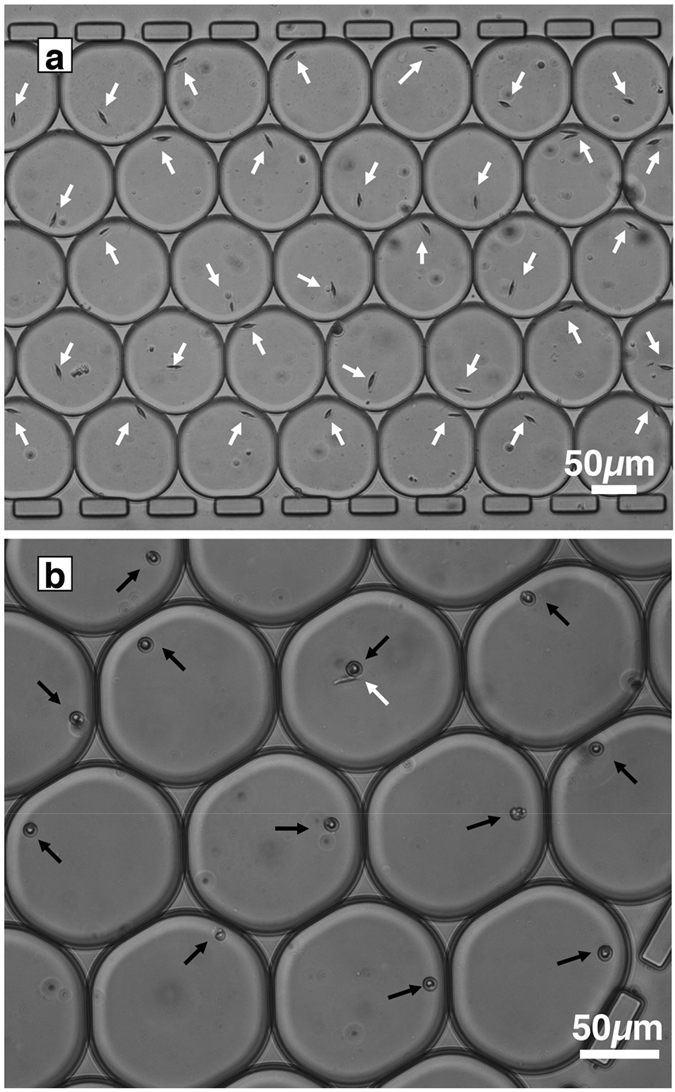
Example of a dual sorting experiment. The top panel (**a**) shows a part of collection channel 1 containing *Phaeodactylum tricornutum* encapsulated in droplets. The bottom panel (**b**) shows a part of collection channel 2 containing *Dunaliella tertiolecta* in droplets. White and black arrows show the location of *P. tricornutum* and *D. tertiolecta* in the droplets, respectively. The white arrow in panel b shows that two different species were used in a single run.

To determine the efficiency of both recognition and sorting of our device, we used two plankton species (*D. tertiolecta* and *P. tricornutum*). A mixture of these two plankton species was emulsified, and droplets containing single planktons were sorted depending on the plankton morphologies (Video 1). The results indicated that 90 ± 3.8% and 91 ± 4.5% of droplets in the collection channels contained a single plankton species (*P. tricornutum* and *D. tertiolecta*, respectively) (Fig. 3). The percentage of correct targets not sorted (hardware default) was lower than 0.5% of the total number of droplets of interest observed in the detection area. False targets and a false number of targets were the main sources of error found in the collection channels of *P. tricornutum* and *D. tertiolecta*, respectively. This difference was linked to the difficult detection of the transverse axis of the *P. tricornutum* frustule (<5 μm) despite a low similarity index (70%). This difficult recognition of *P. tricornutum* led to a higher percentage of false cell numbers (8%) in droplets in the channel collecting *D. tertiolecta.* Comparison of the typical morphology of *D. tertiolecta* detected with a similarity index set at 80% was robust to detect this flagellate species (1% false target error). Figure 4 and Video 2 illustrate typical results obtained in collection channels when a dual sorting experiment with a mixture of live cells was performed.

### Plankton incubation in droplets

**Figure 5:**
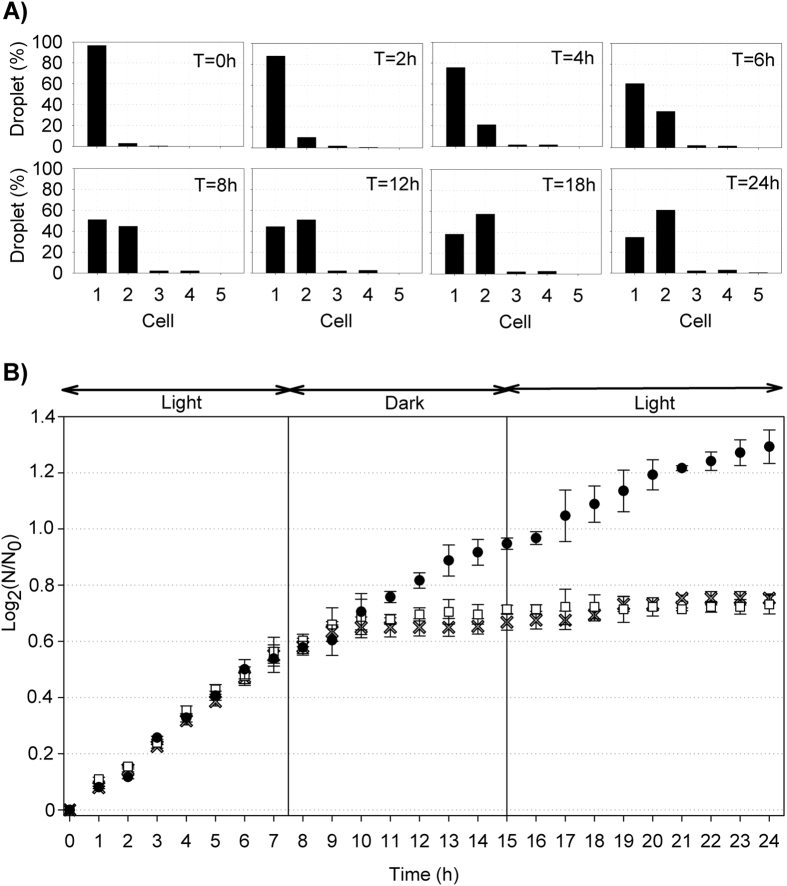
*Phaeodactylum tricornutum* count data in droplets (**A**) and their total abundance expressed as log_2_(N/N_0_) depending on the time (h) (**B**) (n = 3). The gray crosses are the abundance of diatoms incubated in monodispersed droplets (1 nL) after the sorting step (1 cell droplet^−1^). The white squares are diatoms concentrated to 1 cell nL^−1^ and incubated in a large flask (25 mL). The black dots are the abundance of diatoms incubated in a large flask (25 mL, initial concentration: 1 cell μL^−1^).

To examine how long single *P. tricornutum* cells incubated in an emulsion of monodispersed droplets can divide in the chip after the screening process, we conducted incubation experiments for 24 h. The results indicated that the abundance of diatoms in droplets increased at the same rate as in large culture flasks (25 mL) within 9 h following the end of the sorting experiment (Fig. 5). The stationary phase was reached after 9 h of incubation for both species in droplets and in large culture flasks with a high initial concentration (1 cell nL^−1^). The stationary phase occurred in the dark light condition. However, in comparison with Quraishi and Spencer^32^, the effects of dark and light conditions on the division rate of cells were not clearly evidenced over 1 day of the experiment. For example, the total abundance of cells in the large flask with a low initial concentration (1 cell μL^−1^) linearly increased during the entire experiment and reached a division rate of 1.29 ± 0.06 d^−1^. This division rate was in agreement with the literature^33^,^34^. Although the growth rate of cells in droplets was close to that in large flasks during 9 h, major variability was evidenced between each droplet. For example, 34% of single live cells encapsulated in droplets did not divide during 24 h, but 5.5% of cells divided more than once (Fig. 5a). This intra-species variability in growth rate was observed with difficulty in large flasks, but the results indicated that it could be easily monitored using the microfluidic technology and time lapse analyses.

## Discussion

We report the development of a new microfluidic platform to screen droplets and incubate live plankton species at the single cell level. Selection of droplets of interest is performed using a combination of two original image-processing algorithms exploring plankton geometry as the sorting criteria. This label-free technique takes advantage of the diversity of plankton morphologies to sort target species from a mixture of cells. This approach differed from advanced image recognition systems such as snake, background subtraction-based algorithms, and the recent combination of forward and side scatters in fluorescence-activated cell sorting sorters that mainly detect objects^14^,^35^,^36^,^37^,^38^. The major advantage of this technology compared with other methods is the possibility to recognise any object in a droplet without fine-tuning parameters or an extensive data set to calibrate the system^37^. Because of the high abundance of particles (e.g. particulate inorganic and organic matter), recognition of an object is crucial for marine sample analyses. Lacking this recognition step in the image-processing algorithm can lead to an increase in the false positive signal due to particles with the same projected area as the target. The ability to recognise cells in a heterogeneous sample demonstrates that the developed template-matching algorithm can be used to directly track species encapsulated in droplets. The rotating search implemented in the template-matching algorithm also provides the advantage of recognising objects that are not oriented in the axis of flow and is not limited to round objects^39^,^40^,^41^. Because of the pixel level resolution of the image-processing algorithms, recognition of cells in each image required a higher processing time and led to a lower sorting rate than in the literature (SM1). However, future implementation of the algorithms into a field programmable gate array, (FPGA) could significantly decrease processing time. An FPGA is particularly suitable to process high speed calculations without the common limitation of the operating system^42^,^43^. Because processing speed represents the longest portion of the calculation time, future applications using an FPGA device could improve the screening speed of droplets.

Screening droplets of interest was performed using an original liquid electrode design. In comparison with conventional electrodes reported in the literature, the advantages of the saline solution in the electrode channels are: (*i*) the absence of contact between the main channel and electrodes^19^,^21^; (*ii*) conducting direct current (DC) pulses of high voltage (up to 1,500 V) without any damage observable on the electrode^44^; (*iii*) creating the electrode channel using a conventional chip fabrication protocol^45^; (*iv*) the cost of saline preparation is cheaper than low melting temperature metal alloy or micro–nanoparticles of metal^9^,^46^; (*v*) setting and filling processes of the four electrodes can be performed in less than 5 min prior to analysis. The main disadvantage of the saline solution liquid electrodes compared with agarose liquid electrodes is the possible formation of microscopic NaCl crystals in the electrode channels in the case of long experiments or drying by Joule heat. To prevent the formation of any NaCl crystals by water pervaporation across the PDMS membrane, the chip can be confined to a humidified incubator during the experiment^47^,^48^.

Although hydrodynamic cell trapping or single cell printing systems have been used for single cell isolation and culture, common systems reported in the literature are dedicated to non-motile cells^49^,^50^,^51^,^52^,^53^,^54^,^55^,^56^ (SM4). However, flagellate species are commonly found in seawater samples and they can escape hydrodynamic traps. In particular, we have reported that they automatically swim at the counter current in a chip channel. These rapid swims observed with flagellate species may be a survival strategy developed to escape from predators^57^,^58^. Hydrodynamic cell-trapping designs are also mainly limited to the size of targets. For example, large traps can isolate clustered cells, and sub-micro features have been reported to trap single bacterial cells^56^,^58^. Because of the diversity of plankton morphologies, these highly size-selective traps are most likely not suitable for unbiased single plankton isolation from a heterogeneous mixture. Therefore, encapsulation of plankton species in a size-tuneable droplet appears to be a more suitable solution to investigate a wide range of species including those with motile cells.

To summarise, we have developed an integrated microfluidic system capable of encapsulating live planktons and sorting droplets depending on the cell morphologies within the droplets. The integrated microfluidic design enables both screening and incubation of cells encapsulated in droplets. The results indicated that two plankton species with different morphologies can be discriminated and trapped in collection channels. Our results also indicated no significant difference in the division rates of *P. tricornutum* between nanoliter droplet incubation and large flask chambers for up to 9 h. The advantage of the present method makes it possible to isolate a single live target species from a mixture of cells and precisely follow their behaviours at the single cell level. The developed method also revealed intra-species variability in growth rates at the single cell level. This variability observed with difficulty in large flasks can be easily monitored using this microfluidic technology and time lapse analyses. By focussing on the single cell level, a microfluidic approach offers additional advantages for chronobiology studies commonly restricted to the community level in oceanography.

## Methods

### Cell-sorting chip

The cell-sorting chip (Fig. 1) was prepared from PDMS by soft lithography^59^. Using an ultraviolet exposure contact (Mikasa Corp, MA-20) through a photolithography mask, a mold of SU-8 was created on the silicone wafer (Microlithography Chemical Corps). After placing the PDMS base on the mold, it was heated at 90 °C for 1 h using an ESPEC ST-110 chamber. Then, the PDMS was peeled off the mold, and the input and output ports were punched using a 2.4 mm diameter drill. Small pieces of PMDS extracted with a drill were cleaned using a clean air duster. The PDMS was fixed to a 40 × 50 × 0.4 mm glass slide by exposing both parts to oxygen plasma (30 sec at 50 W; Compact Etcher FA-1, Samco Int. Res. Center, Kyoto, Japan) and gently pressed them together. A surface treatment solution [5 v/v% 1H, 1H, 2H, 2H-(heptadecafluorodecyl) trichlorosilane (Wako, 356-28131)] was used as a water repellent to maintain droplet generation during the experiment. Prior to analysis, the surface treatment solution in pure hydrofluoroether HFE-7300 (Novec) was injected from the oil inlet in the microfluidic devices. To optimise the distribution of the surface treatment solution near the collection channel, negative pressure was applied to the discarded outlet. One minute following injection of the surface treatment solution, an excess of the surface treatment solution was gently collected using a sterile pipette and discarded. Then, the microfluidic channels were immediately rinsed using the pure HFE-7300 reagent and Pico-Surf I (2% surfactant in fluorinated oil FC-40, Dolomite).

### Droplet formation and vision system

The dual pump system consisted of two independent compact air cylinders (Misumi, MSCCN50-50). The pressure of each cylinder was adjusted using a set of air pressure sensors (Keyence, AP-C30 series) and a screw fixed to the hydraulic cylinders. The dual pump system was directly connected to the chip using two separate clean plastic hoses. This dual pump system was used to generate droplets by flow focussing an aqueous stream with two streams of fluorinated oil containing a surfactant (2% Pico-Surf I). Six different flow patterns were observed during the experiment. Sample only and oil only patterns were observed when the sample or oil filled the main channel, respectively. Annular flow was an intermediate state between the plug and sample only patterns, and was found when oil and sample pressures were higher than 2.5 and 4.5 KPa, respectively. The plug pattern occurred when droplets sizes were larger than the main channel (140 μm, SM2a). Droplet frequency and size were measured after stabilisation of the flow type in the channels (<10 min). The time needed for stabilisation of the flow type was maximum at the limit between the droplet and oil flow only patterns. The optical setup consisted of an IX71 Olympus inverted microscope equipped with a set of neutral density, light-balancing daylight (LBD) and green (IF550) filters. Observations were performed using a 400× magnification objective lens. A high sensitivity colour complementary metal-oxide-semiconductor camera (Thorlabs GmbH, DCC3240C) was mounted on the side camera port of the microscope to capture digital images during droplet formation and sorting. This camera was connected to a computer with LabVIEW software and Vision module.

### Cell recognition and image-processing optimisations

Cells encapsulated in droplets were detected using a first algorithm^29^. This algorithm consisted of (*i*) detection of the edge of a cell with complex morphology using a wavelet representation, (*ii*) reduction of the noise and halo, and (*iii*) linking the different parts of an edge to form a recognisable cell shape. This simplified image of a target cell was learned by the computer as a template image before a sample run. During the sample analysis, a second image-processing step, named the template-matching algorithm, was used to search in each image captured by the camera in the presence of learned templates^30^. Using a threshold value, the template-matching algorithm extracted geometric information from the template image (location of seed points, edge) and stored the information during the learning phase. Then, during the matching stage, the algorithm found matches by locating regions in the inspection image, where features aligned themselves in spatial patterns similar to the spatial patterns of the features in the template.

Similarity between a template and image (%) was used to identify to which extent the inspected region in the image matched the template. This parameter was calculated during the matching stage and could be compared with a threshold percentage to improve the identification accuracy. Consequently, the target object or cell was assumed to be present when the similarity (%) measured in the inspection image was higher than the similarity threshold. The similarity was calculated as a standard Euclidian distance between two sectors as follows: Considering a template image *t*(x, y) of size KxL within and image *f*(x, y) of size MxN where K ≤ M and L ≤ N. The cross correlation between *t*(x, y) and *f*(x, y) at a point (*i, j*) is given by the equation (1):


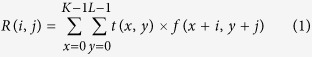


The cross correlation can be scaled so that it lies in the range 0–100. Thus, the cross correlation may be redefined as a similarity in percentage, S(*i, j*) as equation (2):


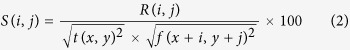


To optimise detection and identification of a single cell, three different templates of the same species were routinely used for each camera frame^31^. A comparison of template locations was added to the template-matching algorithm to prevent multiple counts of a cell. In this study, the distance between the center of a cell detected with the first template image and the centers of the cell detected with the two other templates lesser than 10 pixels was assumed to be only one cell. The distance of 10 pixels (i.e. 1 μm) was chosen according to the diameter of the plankton used in the sorting experiments. A second optimisation was based on controlling the number of target cells detected during a fixed time. This method consisted of starting a cool down timer (50 ms) when the total number of targets was higher than desired. During the cool down, the DC pulses were stopped and droplets could not deviate in any collection channels.

### Droplet sorting

When both morphology and the cell number in an image were the same as the settings, the image was automatically stored in a file and a transistor-transistor logic (TTL) signal was sent to an electronic network (Fig. 2). The electronic network consisted of a data acquisition system (DAQ, National Instrument USB-6009) and original electronic device. Depending on the TTL signal, the data acquisition instrument outputted a DC voltage of 5 V to the original electronic network. This electronic network, consisting of a series of solid state relays, connected the high voltage amplifier (Trek, Model 2220) to the electrode channels of the chip. Then, a high voltage tension (50 ms, −800 V) was delivered to the chip using electrode channels previously filled with saline (0.5 M NaCl). This tension deflected the droplet of interest into a selected collection channel. According to the electrode channel selected with the electronic network, two different targets could be sorted during the same experiment.

### Plankton cultures and incubation

The chlorophyceae *D. tertiolecta* and diatom *P. tricornutum* were cultivated in F/2 medium at 22 °C^60^. These species were selected according to their different morphologies and because their sizes were smaller than the channel depth (25 μm). A mixture of equal concentrations of these two species was used to examine the efficiency of cell discrimination of the microfluidic sorting system.

Three different incubations of *P. tricornutum* (droplets, high and low initial concentrations of diatoms) were conducted to compare the growth of the diatom between large volume incubation chambers (25 mL) and droplets (1 nL) sorted in the chip. Droplets containing cells were sorted and trapped in a collection channel according to the method described above. Incubation of the high initial concentration of the diatom was performed in large sterile flasks (25 mL) by adjusting the concentration to 1 cell nL^−1^. Incubation of the low initial concentration of diatom (1 cell μL^−1^) was considered as conventional cell culture. Both large flasks and chips were stored in the same conditions (light and temperature) during the incubation time (24 h). The abundance of cells in droplets and large flasks were measured stepwise every 30 min by counting the cells in each droplet trapped in the collection channel and using Sedgewick rafter counting chambers, respectively. Incubation experiments were conducted in triplicate.

### Measurement of the swimming speed of *D. tertiolecta*

The swimming speed of *D. tertiolecta* was measured in the sample channel using the template-matching algorithm and a sterile microfluidic chip. Samples consisting of a mixture of *D. tertiolecta* and 5 μm polystyrene beads were injected into the sample inlet, and then the chip was connected to the dual pump system. The 5 μm diameter beads were used to evaluate the speed of aqueous phase flow in the sample inlet. The speed of beads flowing in the sample channel was calculated by measuring the distance and angle between two successive locations of the template identified in the image. Using a laptop computer, calculations of the speed and angle of an object flowing in the channel were performed at 33 Hz (30 ms). The swimming speed of a cell was then obtained by considering the direction and speed of the flow in the sample channel.

## Additional Information

**How to cite this article**: Girault, M. *et al*. An on-chip imaging droplet-sorting system: a real-time shape recognition method to screen target cells in droplets with single cell resolution. *Sci. Rep.* **7**, 40072; doi: 10.1038/srep40072 (2017).

**Publisher's note:** Springer Nature remains neutral with regard to jurisdictional claims in published maps and institutional affiliations.

## Supplementary Material

Supplementary Video 1

Supplementary Video 2

Supplementary Information
