# Supplementary material for: An on-chip imaging droplet-sorting system: a real-time shape recognition method to screen target cells in droplets with single cell resolution
Source: Sci Rep. 2017 Jan 6;7:40072. doi: 10.1038/srep40072 (PMC5216404; doi:10.1038/srep40072)
Supplement: Supplementary Information [file srep40072-s3.doc]

**An on-chip imaging droplet-sorting system: a real-time shape recognition method to screen target cells in droplets with single cell resolution**

**Mathias Girault, Hyonchol Kim, Hisayuki Arakawa, Kenji Matsuura, Masao Odaka, Akihiro Hattori,**

**Hideyuki Terazono, Kenji Yasuda^*^**

**Supplementary material table 1**: List of main encapsulation or sorting droplet methods reported in the literature. *na.* is the information not mentioned in the paper.

|  | **Encapsulation and/or sort types** | **Sorting using fluorescence** | **Sorting depending on the shape** | **Sorting depending on the number of cell encapsulated** | **Multiple sorting** | **Nb. of chip** | **Frequency (Hz)** | **Efficiency** | **References** |
| --- | --- | --- | --- | --- | --- | --- | --- | --- | --- |
| **Passive** | Hydrodynamic | No | No | No | No | 1 | 160 | 70-80 % | 4 |
|  | Inertial | No | No | No | No | 1 | ~8.000 | ~80 % | 5 |
|  | Dean-force | No | No | No | No | 1 | 2.700 | 77 % | 6 |
| **Active** | Poisson’s law,  AC voltage | Yes | No | No | No | 2 | 300 | 99.9 % | 9 |
|  | Poisson’s law,  AC voltage | Yes | No | No | No | 2 | 200 | *na.* | 10 |
|  | Poisson’s law,  AC voltage | Yes | No | No | No | 2 | 29.000 | 99.3 % | 11 |
|  | Poisson’s law,  AC voltage | Yes | No | No | No | 2 | 2,000 | *na.* | 12 |
|  | Poisson’s law,  AC voltage | Yes | No | No | No | 2 | ~9 | *na.* | 13 |
|  | Poisson’s law, Membrane valves | Yes | No | No | No | 2 | 250 | >99.99 % | 14 |
|  | Poisson’s law, Solenoid valves | Yes | No | Yes | No | 1 | 5 to 30 | 87-95 % | 15 |
|  | Poisson’s law,  Solenoid valves | Yes | No | No | No | 1 | *na.* | 94.1 % | 16 |
|  | Acoustic waves | *na.* | No | *na.* | Yes | 1 | *na.* | *na.* | 17 |
|  | Poisson’s law,  DC voltage using  liquid electrodes | Possible | Yes | Yes | Yes | 1 | 10 | Depending on the target  90 ± 3.8 %  91 ± 4.5 % | This study |

**Supplementary material 2**: Size (µm) and frequency (droplet per second, Hz) of droplet depending on the sample and oil pressures. Six flow patterns were indicated in order to set the suitable range of pressures needed to generate stable mono-disperse droplet flow using the chip.

**Supplementary material 3**: Swimming speed (µm s^-1^) of *Dunaliella tertiolecta* measured in the sample channel (2,500 measurements).

**Supplementary material 4:** List of main isolation techniques using microfluidics in the literature.

| **Single cell**  **isolation** | **Detection of a target** | **Recognition of a target** | **Morphology-based**  **technique** | **References** |
| --- | --- | --- | --- | --- |
| Hydrodynamic trap | No | No | Yes | 49, 51, 61, 62 |
| Single cell printer | Yes | No | Yes | 37, 63, 64, 65 |
| Well | Yes | No | No | 35 |
|  | No | No | No | 66, 67 |
| Droplet-based | Yes | Yes | Yes | This study |

**Video 1:** Plankton sorting.

**Video 2:** Typical result obtained in collection channels when a dual sorting experiment with a mixture of live cells.
